# A Statistical Model to Assess Risk for Supporting COVID-19 Quarantine Decisions

**DOI:** 10.3390/ijerph18179166

**Published:** 2021-08-31

**Authors:** Sonja Jäckle, Elias Röger, Volker Dicken, Benjamin Geisler, Jakob Schumacher, Max Westphal

**Affiliations:** 1 Fraunhofer Institute for Digital Medicine MEVIS, 23562 Lübeck, Germany; 2 Fraunhofer Institute for Industrial Mathematics ITWM, 67663 Kaiserslautern, Germany; yukio.elias.roeger@itwm.fraunhofer.de; 3 Fraunhofer Institute for Digital Medicine MEVIS, 28359 Bremen, Germany; volker.dicken@mevis.fraunhofer.de (V.D.); benjamin.geisler@mevis.fraunhofer.de (B.G.); max.westphal@mevis.fraunhofer.de (M.W.); 4 Health Department Berlin-Reinickendorf, 13407 Berlin, Germany; jakob.schumacher@reinickendorf.berlin.de

**Keywords:** COVID-19, decision support, bayesian statistics, quarantine, risk assessment

## Abstract

In Germany, local health departments are responsible for surveillance of the current pandemic situation. One of their major tasks is to monitor infected persons. For instance, the direct contacts of infectious persons at group meetings have to be traced and potentially quarantined. Such quarantine requirements may be revoked, when all contact persons obtain a negative polymerase chain reaction (PCR) test result. However, contact tracing and testing is time-consuming, costly and not always feasible. In this work, we present a statistical model for the probability that no transmission of COVID-19 occurred given an arbitrary number of negative test results among contact persons. Hereby, the time-dependent sensitivity and specificity of the PCR test are taken into account. We employ a parametric Bayesian model which combines an adaptable Beta-Binomial prior and two likelihood components in a novel fashion. This is illustrated for group events in German school classes. The first evaluation on a real-world dataset showed that our approach can support important quarantine decisions with the goal to achieve a better balance between necessary containment of the pandemic and preservation of social and economic life. Future work will focus on further refinement and evaluation of quarantine decisions based on our statistical model.

## 1. Introduction

### 1.1. COVID-19 Pandemic and Situation in Germany

Since spring 2021, COVID-19 spread to almost all countries around the world [1]. Many countries legislated strict lock-downs in order to decrease infections numbers. When the infection numbers have reached an acceptable low level, more liberal rules applied, such as social distancing and limits on larger gatherings. In addition, many countries have issued regulations requiring infected persons to be isolated and contact persons to be quarantined. In Germany, local health departments report information about their local, new cases daily to the Robert Koch Institute (RKI), the central German institution for infectious disease study and control. The RKI reports on a regular basis about the COVID-19 situation in Germany; see, for example, Reference [2]. Based on those reported numbers, the RKI recommends an implementation, adaption, or cancellation of procedural rules for political decision-makers in Germany.

Since August 2020, a second significant increase of new infections per week has been reported in Germany, as well as in many other European countries. Many of these cases were associated with vacation travels. In Germany, it resulted in several times higher numbers than reported in June, where the number of new infections per day had reached a minimum so far [3]. This triggered strong societal discussions about risks involving the reopening of schools and re-allowing events, such as mass gatherings and shortening of the quarantine duration time for contact persons. This infection increase resulted in the so-called second wave in end of 2020, which continued until the start of 2021. Due to the spread of the alpha variant, the number of infections increased again in February and triggered a third wave of infections in spring of 2021. A similar situation occurred in summer 2021. Since June 2021, the delta variant, which already dominated the infections in July, has caused a further increase in infections.

### 1.2. Quarantine Decisions in Germany

The German Protection against Infection Act (“Infektionsschutzgesetz”) [4] defines the general rules for dealing with infectious diseases. It specifies which pathogens must be monitored and allows German health departments to quarantine people at home. In the current COVID-19 pandemic, all contacts with close contact to the infected person and, therefore, at high risk of infection have to stay at home. Since a large number of COVID-19 cases was reported by the RKI, a large number of contact persons probably had to be quarantined in Germany [2]. The RKI provides the procedural guidelines for theses cases. In the end, however, the local health departments have to decide autonomously whether and for how long a person or a group of persons have to be quarantined.

This is the first time in many decades that health departments have had to quarantine such a large number of infected individuals and contacts. In recent decades, quarantines have been imposed only a few times. For example, in 2002/2003, the first severe acute respiratory syndrome (SARS-CoV) started to spread over several countries. In Germany, contact persons were quarantined at home, but the number of quarantined was substantially smaller than in the current COVID-19 pandemic [5]. A default workflow for contact tracing of COVID-19 cases is provided by the RKI [6]. All contact persons are classified into one of the three categories, that are listed in Table 1.

The RKI defines category 1 as persons who

- had face-to-face contact for at least 15 min or
- had direct contact to bodily fluids or
- were exposed to a relevant aerosol concentration.

Other contact persons are classified as category 2. Medical staff with appropriate personal protective equipment is classified as category 3. The RKI recommends that contact persons of category 1 and 2 normally should reduce their contacts to other persons, and contact persons of category 1 additionally should stay quarantined at home until 14 days after exposure. Moreover, contact persons of category 1 should be tested, ideally one day after case documentation and a second time 5–7 days after contact with the COVID-19 case [6].

Since February 2020, a high number of contact persons of COVID-19 had to stay in home quarantine, especially during the infection waves, when the highest number of active COVID-19 cases occurred. The RKI collected data about all COVID-19 spreads until 11 August 2020 and reported the frequency and size for different event types [7]. The probability of a transmission and the number of infected people depends noticeably on the event type. As of 11 August 2020, 3902 spreads occurred in households and 709 spreads in retirements homes, whereas only 31 spreads were reported at schools and 33 at kindergartens [7].

This indicates that the risk of a COVID-19 spread in a group depends strongly on the scenario. This fact might inform quarantine decisions and might justify more liberal quarantine decisions in certain situations. However, the event type is currently not taken into account systematically according to the RKI recommendations. Furthermore, it is often not possible to get test results from all participants of a contact event. Reasons for this might be that testing capacity is limited, the persons could not be contacted at all or they did not go to a test center.

Moreover, quarantine can indirectly cause economic damage. Since the persons have to stay at home or employees have to take care of their quarantined children, they are unable to work during the quarantine. In addition, quarantines of persons, which work in system relevant positions, can lead to an undersupply of critical infrastructure. This includes medical care and food supply, but also other sectors, such as government administration and supply of water and energy.

Against this background, it is desirable to support these quarantine decisions with objective data and statistical models because many people are involved in the process. A software implementing such a model can streamline and support the decision making process and make it less prone to human mistakes. In literature, different models have been proposed for explaining COVID-19 transmission dynamics [8,9] or predicting the number of COVID-19 infections [10,11]. Those models allow the governments to adapt their COVID-19 regulations in order to control the COVID-19 pandemic. However, it also important to directly support the institutions which are responsible for monitoring the COVID-19 infected persons in their district. In literature, a statistical model for describing COVID-19 transmissions of an infected person [12] and a model for estimating the infection risk in meetings [13] has been introduced. However, no model for decision support of group quarantines has been reported, to the best of our knowledge. Thus, this is the first work which describes a statistical model as decision support for group quarantines.

### 1.3. Structure of the Paper

In this work, we will provide a statistical model for the risk assessment of canceling group quarantines. It allows to estimate the risk of canceling a group quarantine, where a fraction of the group has been tested and all test results are negative. In Section 2, the Bayesian model including the prior choice is described. The proposed model can be used for any group event situation in any country. In this work, it is applied for a school class scenario and analyzed in Section 3. Here, the model specified for school classes was tested with a real-world data-set of a German school class. Finally, the model and the results are discussed in Section 4.

## 2. Materials and Methods

### 2.1. Use Case

In the following, we describe the idealized use case for our model. We assume that a group contact event took place, e.g., a family party, a sports event, or a teaching unit in school. A certain time after the event, one or multiple participants are tested positive for COVID-19, possibly only after the onset of symptoms. More generally, a series of contact events with the same group (e.g., in school or at work) might have taken place. We presume that there is only a single person known to be infected, the so-called *primary* or *index case*. Adaptations for multiple primary cases are discussed in Section 4. The remaining group, without counting the primary case, is assumed to consist of *M* individuals with unknown infection status. As a precautionary measure, this group of potential *secondary cases* is quarantined by health authorities to contain further infection dynamics. In addition, all contact persons are invited to take a polymerase chain reaction (PCR) test. Currently, all contacts should stay in quarantine for 14 days [14].

**Figure 1:**
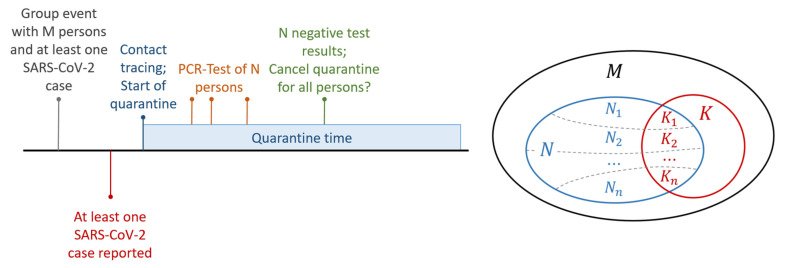
**Left**: Timeline of the events starting from the group event until the quarantine decision. **Right**: Overview of the group with *M*, the possible number of infected *K*, and the negative tested persons *N*. For *N* and *K*, the contact persons are divided into subgroups $N_{d}$ and $K_{d}$, which are tested on day $D_{d},d\in \left\{1,\ldots ,n\right\}$, are also illustrated.

We are now interested whether there occurred any transmissions of COVID-19 from the primary case to any of the contact persons during the group event. Any other transmission paths outside of the group event are not considered. If there is at least one positive test result in the remaining group, this indicates that a COVID-19 transmission occurred. In this case, the health offices in Germany are not allowed to cancel the group quarantine because other persons might be also infected, and this has to be checked. Thus, the case of interest is that all test results are negative. Figure 1 (left) illustrates the timeline of these events described above. The main question which guided the methodological developments in this work is:

“Under which conditions can a group quarantine be released (earlier) such that the probability of overlooking a (secondary) infection is low?”

In an ideal world, all potential cases would be tested by a highly accurate test. However, we rather suppose that not all *M* individuals are tested due to resource constraints or other reasons. Instead, out of the *M* person group, N≤M are successfully tested negative.

In our situation, we consider reverse transcription PCR tests with (time-dependent) imperfect accuracy. An extension to other tests, as, for example, the rapid antigen test, is possible as long as the specificity and sensitivity can be characterized. Thus, the probability that all test results are negative, even though there are some infected persons in the group, is not zero. As a further complication, we allow that not all *N* tests are conducted on the same day (after the group contact event). Rather, usually, test time points vary due to logistic or resource limitations. In summary, we have only partial (probabilistic) knowledge regarding the disease status of the remaining group. The above question, thus, can be formulated more precisely as:

“How likely is it that there is no secondary infection, given all *N* out of *M* people are tested negative?”

The model described in the following assumes that the people to be tested are chosen randomly. This is not likely to hold in the real world as most authorities will give priority to testing people that were close spatially or socially to the primary case(s). As a result, it is less likely that an individual with a high risk gets released from quarantine without a test. If that is the case, the model will slightly overestimate the risk of an infection. In consultation with the health office Berlin-Reinickendorf, we concluded that this calculation is appropriate.

### 2.2. Statistical Model

The goal of the model is to estimate the probability, that no COVID-19 transmission occurred. As outlined above, we consider *M* contact persons, N≤M of them having been tested and all tests results are negative. Moreover, we have an unknown number of infected persons K;K≤M. We consider only the secondary transmissions caused by the group event, but no further potential infections outside of the participant group *M*. Figure 1 (right) gives an overview over these (sub)groups. According to Bayes rule, the posterior distribution for a given number of K=k is then described as

(1) $$\begin{array}{cc} \; & P(K=k|N\;\mathrm{out}\;\mathrm{of}\;M\;\mathrm{tested}\;\mathrm{negative}\;\mathrm{at}\;\mathrm{specific}\;\mathrm{days})\\ \; & =\frac{P(N\;\mathrm{out}\;\mathrm{of}\;M\;\mathrm{tested}\;\mathrm{negative}\;\mathrm{at}\;\mathrm{specific}\;\mathrm{days}|K=k)\cdot P(K=k)}{P(N\;\mathrm{out}\;\mathrm{of}\;M\;\mathrm{tested}\;\mathrm{negative}\;\mathrm{at}\;\mathrm{specific}\;\mathrm{days})}. \end{array}$$

For our use case, we are interested in the probability, that no one is actually infected (K=0) given that *N* out of *M* have been tested, and all tests result are negative. This probability is then given as

(2) $$\begin{array}{cc} p_{0} & :=P(K=0|N\;\mathrm{out}\;\mathrm{of}\;M\;\mathrm{tested}\;\mathrm{negative}\;\mathrm{at}\;\mathrm{specific}\;\mathrm{days})\\ \; & =\frac{P(N\;\mathrm{out}\;\mathrm{of}\;M\;\mathrm{tested}\;\mathrm{negative}\;\mathrm{at}\;\mathrm{specific}\;\mathrm{days}|K=0)\cdot P(K=0)}{P(N\;\mathrm{out}\;\mathrm{of}\;M\;\mathrm{tested}\;\mathrm{negative}\;\mathrm{at}\;\mathrm{specific}\;\mathrm{days})}\\ \; & =\frac{P(K=0)}{\sum_{i=0}^{M}P\left(N\;\mathrm{out}\;\mathrm{of}\;M\;\mathrm{tested}\;\mathrm{negative}\;\mathrm{at}\;\mathrm{specific}\;\mathrm{days}|K=i\right)P\left(K=i\right)}, \end{array}$$

since it is assumed, that healthy persons are always tested negative, which is described in more detail in Section 2.4. Thus, P(NoutofMtestednegative|K=0)=1 holds.

This requires an a-priori probability distribution P(K) for the number of infected persons *K*. This prior describes which values of *K* are considered probable before considering the data. Furthermore, a likelihood function P(NoutofMtestednegative|K) is needed, which describes the probability distribution that *N* of *M* are tested negative under the condition that *K* persons are infected in the group. The modeling of the prior and the likelihood function are described in the following sections.

### 2.3. Prior Distribution

Transmissions of COVID-19 have a high heterogeneity, which means that most infected do not infect anybody else whereas some infected persons are causing super-spreading events. Lelieveld et al. [13] introduced a model based on the binomial distribution for SARS-CoV-19 aerosol transmission risk calculation. The negative binomial distribution was employed in the literature to model heterogeneity in secondary infections [12]. However, the negative binomial distribution is defined for an unrestricted support {0,1,2,…}, which is not fitting for a finite (and usually small) group size as considered in this work. We employ a Beta-binomial prior instead, which is a direct extension to the binomial prior. The uncertainty regarding the transmission probability *p* can be modeled using the Beta-binomial as prior distribution. The Beta-binomial distribution is defined as

(3) K|α,β,M∼BB(M,α,β),

with shape parameters α and β. It is a discrete probability distribution on a finite support of non-negative integers, and it is frequently used when the success probability in each of the known number of trials is either unknown or random. Its probability mass function is defined as

(4) P(K=k)=MkB(k+α,M−k+β)B(α,β),

where B(α,β) is the beta function. The probability that the contact persons become infected and the number of infected persons depends on the type of event, e.g., if it takes place outside or inside and how much the persons interact and talk. Thus, the parameters of the Beta-binomial distribution should be adjusted to the considered use case.

In order to define α and β, we use two other parameters for the scenario specific modeling which allows for easier interpretation. We consider the probability that a transmission of COVID-19 to at least one person takes place

(5) P(K>0),

and the conditional expected value

(6) $$E\left(K|K>0\right)=\frac{\sum_{i=1}^{M}P\left(K=i\right)\cdot i}{P(K>0)}.$$

With these two known values, the needed parameters α,β can be determined by solving an optimization problem, for example, with the L-BFGS method [15].

### 2.4. Likelihood

**Figure 2:**
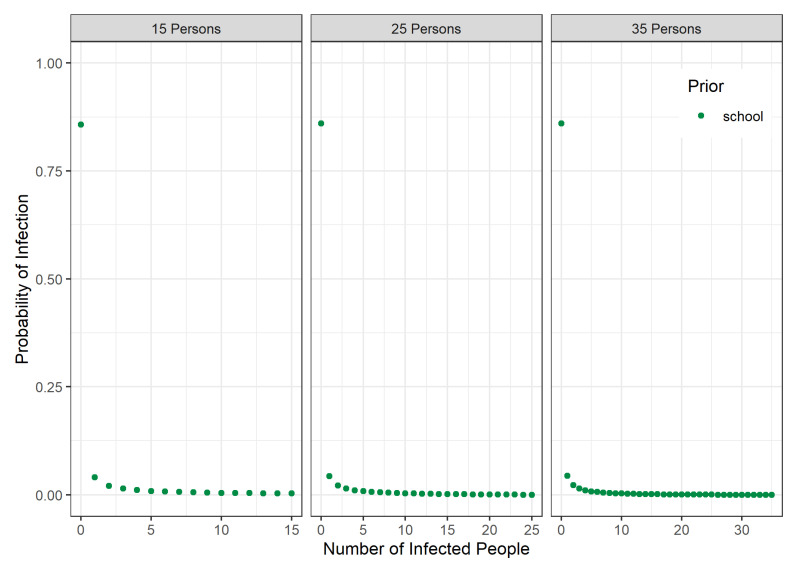
Plots of the Beta-binomial distribution as school class prior for $M=15,25,\begin{array}{c} 35 \end{array}$.

The accuracy of a test is described by the specificity (Sp, also called *True Negative Rate*) and the sensitivity (Se, also called *True Positive Rate*). In this paper, PCR tests are considered for testing. The U.S. Food and Drug Administration analyzed PCR tests and determined a specificity of nearly 100% [16] (the very few false positives are usually caused by laboratory handling errors). Thus, we assume a specificity of 1. However, the sensitivity is less than 1, and the probability that a person infected with COVID-19 is tested positive depends on the time point of testing; see Reference [17], as well. For our model, these probabilities were read and copied manually from the first graph of Figure 2 of publication [17]. Thus, the test of an infected person will be negative with almost 100% probability on the first and second day after the infection; between days six to ten after infection, this probability of a false negative test result drops below 25%, reaching a minimum of about 20% on day eight.

This day specific sensitivity is used for our realistic modeling of the likelihood function: The *N* tested persons are split in *n* day specific groups. $N_{1}$ persons were tested on day $D_{1}$ after the group event, $N_{2}$ persons were tested on day $D_{2},D_{2}\neq D_{1},\ldots ,$ and $N_{n}$ persons were tested on day $D_{n},D_{n}\neq D_{d}\;\mathrm{for}\;d\in \left\{1,\ldots ,n-1\right\}$. In the same way, the *K* infected persons are divided into days: $K_{1},\ldots ,K_{n}$. For both the tested persons $N_{d}$ and the infected tested persons $K_{d}$, we assume that they are only tested on a single day. Thus, every person $x\;\mathrm{is}\;\mathrm{tested}\;\mathrm{on}\;\mathrm{day}\;D_{i}\;\mathrm{and}\;\mathrm{is}\;\mathrm{not}\;\mathrm{tested}\;\mathrm{on}\;\mathrm{day}\;D_{j}\;\mathrm{and}\;i\neq j\;\forall \;i,j\in \left\{1,\ldots ,n\right\}$. The remaining untested and infected persons $N_{0}=M-N,K_{0}=K-\sum_{d=1}^{n}K_{d}$ are considered as an additional group. These subsets of *N* and *K* are also illustrated in the right image of Figure 1. Moreover, the day dependent sensitivity $s_{D_{d}}$ is known for all days $D_{d},d\in \left\{1,\ldots ,n\right\}$.

The distribution of COVID-19 infected persons *K* can now be considered as a random drawing from an urn containing *M* persons divided into the subsets $N_{0},N_{1},\ldots ,N_{n}$. Then, the probability of one specific draw $\tilde{K}=\left(K_{0},\ldots ,K_{n}\right),\sum_{d=0}^{n}K_{d}=K\;\mathrm{and}\;K_{d}\leq N_{d}\;\;\forall \;d\in \left\{0,\ldots ,n\right\}$ can be calculated with the formula of the multivariate hyper-geometric distribution:

(7) P(K˜=(K0,…,Kn))=∏d=0nNdKdMK.

Furthermore, the probability for a negative test on day *d* is needed. For healthy persons, this probability is 1, since we assume a specificity of 100%. However, for infected persons, this probability depends on the test date:

(8) $$\begin{array}{cc} \; & P(\mathrm{Negative}\;\mathrm{test}\;\mathrm{on}\;\mathrm{day}\;D_{d}|\;\mathrm{person}\;\mathrm{is}\;\mathrm{healthy})=1; \end{array}$$

(9) $$\begin{array}{cc} \; & P\left(\mathrm{Negative}\;\mathrm{test}\;\mathrm{on}\;\mathrm{day}\;D_{d}|\;\mathrm{person}\;\mathrm{is}\;\mathrm{infected}\right)=1-s_{D_{d}}. \end{array}$$

On each day $D_{d}$, $N_{d}$ persons with an unknown number of infected persons $K_{d}\leq N_{d}$ are tested and the likelihood of testing them all negative is given by

(10) $$\begin{array}{cc} P(N_{d}\;\mathrm{negative}\;\mathrm{tests}\;\mathrm{on}\;\mathrm{day}\;D_{d}|K_{d}\;\mathrm{persons}\;\mathrm{are}\;\mathrm{infected}) & ={\left(1-s_{D_{d}}\right)}^{K_{d}}\cdot 1^{N_{d}-K_{d}}\\ \; & ={\left(1-s_{D_{d}}\right)}^{K_{d}}. \end{array}$$

The probability for testing all infected persons on all days negative can be determined by multiplying all day specific probabilities, since the number of negative tests is given for each day; thus, the probabilities for each day are independent:

(11) $$\begin{array}{cc} \; & P(\begin{array}{c} N\;\mathrm{total}\;\mathrm{negative}\;\mathrm{tests}\;\mathrm{with}\;N_{d}\;\mathrm{at}\;\mathrm{each}\;\mathrm{day}\;D_{d} \end{array}|K=\sum_{d=0}^{n}K_{d}\;\mathrm{of}\;N_{d}\;\mathrm{persons}\;\mathrm{are}\;\mathrm{infected})\\ \; & =\prod_{d=1}^{n}P\left(N_{d}\;\mathrm{negative}\;\mathrm{tests}\;\mathrm{on}\;\mathrm{day}\;D_{d}|K_{d}\;\mathrm{of}\;N_{d}\;\mathrm{persons}\;\mathrm{are}\;\mathrm{infected}\right)\\ \; & =\prod_{d=1}^{n}{\left(1-s_{D_{d}}\right)}^{K_{d}}. \end{array}$$

For the next step, we are considering a simple example, where all persons are tested on the same day, and it holds that $N=N_{1},N_{0}=M-N$. In this case, the following draws $\tilde{K}$ can occur:

(12) $$\begin{array}{c} X_{K}=\left\{\left(\min\left(N_{0},K\right),K-\min\left(N_{0},K\right)\right),\ldots ,\left(K-\min\left(N_{1},K\right),\min\left(N_{1},K\right)\right)\right\}. \end{array}$$

Thus, the probabilities for all those possible draws defined in Equation (7) multiplied with the negative testing probabilities defined in Equation (11) must be summed up to obtain

(13) P(NoutofMtestednegativeatspecificdays|K)=∑(K0,K1)∈XKNK0M−NK1MK(1−sD1)K1.

In general, the persons are tested on *n* different days, and we obtain:

(14) $$X_{K}=\left\{\left(K_{0},\ldots ,K_{n}\right)|\sum_{d=0}^{n}K_{d}=K\;\mathrm{and}\;K_{d}\leq N_{d}\;\;\forall \;d\in \left\{0,\ldots ,n\right\}\right\}$$

as set of all possible draws. Again, the probabilities have to be summed up to obtain the probability

(15) $$\begin{array}{cc} \; & P(N\;\mathrm{out}\;\mathrm{of}\;M\;\mathrm{tested}\;\mathrm{negative}\;\mathrm{at}\;\mathrm{specific}\;\mathrm{days}|K)\\ \; & =\sum_{\tilde{K}\;\in X_{K}}P\left(\tilde{K}=\left(K_{0},\ldots ,K_{n}\right)\cap \begin{array}{c} N\;\mathrm{total}\;\mathrm{negative}\;\mathrm{tests}\;\mathrm{with}\;N_{d}\;\mathrm{at}\;\mathrm{each}\;\mathrm{day}\;D_{d} \end{array}\right), \end{array}$$

where $P(\tilde{K}=\left(K_{0},\ldots ,K_{n}\right)\cap \begin{array}{c} N\;\mathrm{total}\;\mathrm{negative}\;\mathrm{tests}\;\mathrm{with}\;N_{d}\;\mathrm{at}\;\mathrm{each}\;\mathrm{day}\;D_{d} \end{array})$ is the probability of testing all persons in one specific realization $\tilde{K}=\left(K_{0},\ldots ,K_{n}\right)$. As in the simple example, the probability for the draw has to be multiplied with the probability, that all infected people are tested negative. Using Equations (7) and (11), we obtain

(16) P(K˜=(K0,…,Kn)∩NtotalnegativetestswithNdateachdayDd)=P(K˜=(K0,…,Kn))·P(NtotalnegativetestswithNdateachdayDd|K=∑d=0nKdpersonsareinfected)=∏d=0nNdKdMK·∏d=1n(1−sDd)Kd=N0K0MK·∏d=1nNdKd(1−sDd)Kd.

Thus, the likelihood function is modeled with the above equations, and the posterior probability can be calculated via Bayes Theorem, as outlined in Section 2.2.

## 3. Result

### 3.1. Prior Distribution for School Classes

We conducted a literature search about the probability of a COVID-19 outbreak in schools. This search was done with Google Scholar with different combinations of the keywords SARS-CoV-2, COVID-19, transmission, school, pupils, education. As a result, we found four studies that analyzed the transmission probability in school classes: a French study of one school [18], an Irish study of all COVID-19 cases in schools [19], an Australian study [20] analyzing different educational scenarios, and a study in Luxembourg [21].

In the Australian study, 15 school classes with at least one COVID-19 infected person were analyzed and for 3 school classes new COVID-19 infections were detected [20]. No transmission of COVID-19 in 6 school classes was detected in the Irish study [19]. In the French study, one outbreak was analyzed in detail, especially the transmissions of COVID-19 in one high school [18] that took place at a time prior to enforcement of strict pandemic hygiene rules. This study analyzed an outbreak at a whole school and not in one single school class. The study in Luxembourg analyzed the primary and secondary transmissions in school; however, no information about transmission probability in school classes was reported [21]. Therefore, we decided not to take the last two studies into account. Based on the two remaining sources, we obtain a first crude estimation of P(K>0). Here, we considered all school classes equally. Then, the sum of all school classes with transmissions was calculated and divided by the sum of all considered school classes:

(17) $$P\left(K>0\right)=\frac{0+3}{6+15}=\frac{3}{21}\approx 0.14.$$

Moreover, the RKI recently published a report about COVID-19 outbreaks in different environments [7]. In this document, an analysis about the average number of infected persons per outbreak was made. For school classes, an average number of 4.8 infected persons was reported, which equals the conditional mean value:

(18) E(K|K>0)=4.8.

With these two values, a unique Beta-binomial distribution can be determined as outlined in Section 2.3. The distributions for school classes with M=15,25,35 are plotted in Figure 2. For all cases, a suitable Beta-binomial distribution could be calculated. Small differences between the distributions can be only observed for the probability values with K>0.

### 3.2. Posterior Probabilities and Decision Support for School Classes

**Figure 3:**
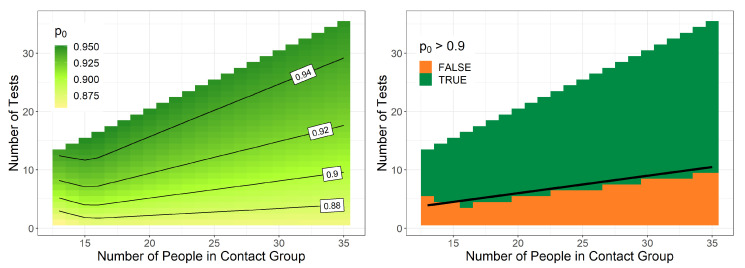
**Left**: Posterior probability that no transmission occurred given that *N* out of *M* persons are tested negative using the specific school prior for different group sizes and number of tests made 4 days after the group event. **Right**: Decision of a quarantine cancellation based the condition that $p_{0}>0.9$. The black line shows the situation that 30% are tested.

In this section, the school class scenario is further considered and the posterior probability is analyzed that no transmission occurred given that *N* out of *M* persons are tested negative. For this analysis, we used the prior specified for school classes which is determined in Section 3.1. In Germany, school classes have usually at least 13 and not more than 35 pupils [22]. Thus, we calculated this probability for different school class sizes M∈{13,…35} and different numbers of tests *N*, which were all done 4 days after the group event. The results are shown in the left panel of Figure 3. This probability ranges from 0.85 up to 1.0. One reason for this high minimum is the specific prior distribution, which should have the probability 0.86 for no transmission. Thus, the posterior probability cannot be smaller than 0.86 due to formula (2). Smaller values are only observed for M<15, where no optimal parameters could be found with the optimization. Moreover, all determined posterior probabilities never reach 100% because there is always some small remaining risk, that there could be some infected persons in the group, who were tested negative.

With this calculated probability, one can make a decision whether the quarantine can be canceled despite the fact that not all persons have been tested. Using, for example, the rule $p_{0}>0.9$, the quarantine will be canceled in most cases, as shown in the right panel of Figure 3. Only if less than around 30% of the group size have been tested is the result $p_{0}$ less than 90%, and the quarantine is continued.

### 3.3. Decision about Quarantine Cancellation for School Classes

The statistical model with the prior specified for school classes (determined in Section 3.1) is tested and evaluated with one dedicated dataset provided by the health department Berlin-Reinickendorf. The dataset is shown in Table 2. We have 17 persons which were in contact with one infected person. All of them had contact on 10 August, for most of them it was the last contact. However, two of them have met the person later on until 16 August and 18 August again. These two persons do not fit into our group event setting and hence were excluded. Employees at the German health departments consider and decide about the quarantine of these persons separately on an individual basis.

**Figure 4:**
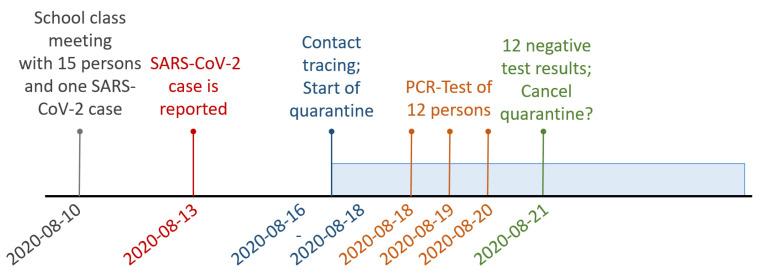
Timeline of the school class example.

As a result, we now have 15 persons with last contact on August 10. Twelve persons were tested, and all test results are negative. One person was tested on August 18, ten persons on August 19, and one person on August 20. The timeline for this sequence of events is given in Figure 4. With this information, we can calculate the posterior probability, that nobody at all was infected according to our model. With the prior distribution determined for a school class with M=15 pupils, we obtain a probability of $p_{0}=98\%$, that no transmission of COVID-19 occurred in this setting. Based on the described decision rule of $p_{0}>0.9$ suggested in Section 3.2, this result would support to cancel the quarantine for the whole group early. Indeed, no further COVID-19 cases were reported to the health department concerning this particular school class. Hence, the correct decision about cancellation of quarantine would have been made in this specific case.

## 4. Discussion

The results show, that the proposed statistical model can support quarantine decisions by risk estimation. The generalizability of our approach to other countries and/or other types of group events is limited and depends on the availability of suitable evidence to inform the prior distribution. For that reason, we would advise to conduct a literature research to derive meaningful prior parameters before adopting our model to other scenarios.

The results from the proposed model depend on the chosen prior distribution which is subject to uncertainty. In Bayesian analysis, a non-informative prior is sometimes phrased as a solution to this problem. We would, however, discourage using a “non-informative” uniform prior in the considered scenario because of epidemiological implausibility: for instance, under a uniform prior, the prior probability of no (K = 0) or all (K = M) contact persons being infected is equal, which we do not consider to be realistic.

Furthermore, general and quite simplified assumptions were made for the different probability distributions. Several aspects, such as the number of initially infected persons in the group or other aspects of the group event (inside/outside, ventilation, activity type, interactions, etc.), were not addressed. A paper was published, which describes a model for estimating the aerosol transmission risk depending on several parameters e.g., room properties, event duration or number of participants [13]. Based on this work, the probability that a transmission of COVID-19 occurred can be estimated. However, further information about the average number of infected persons is not available using this approach introduced in Reference [13]. Currently, there is still a lack of available data and published studies for extending our statistical model. In addition, for the likelihood, we assumed that every contact was tested once. However, normally, contact persons are tested two times.

Since the prior specification is always subjective and depends on the availability of adequate evidence for model parameters, the sensitivity of the results to the prior should be addressed in the future. Different frameworks to approach robust Bayesian analysis have been described in the literature [23]. Furthermore, we assumed a random choice of tested persons, but, in reality, the contact persons with the highest risk would be chosen for tests. The likelihood was specified for the PCR-tests, but this could be also adapted for other test types when the tests sensitivities and specificity are known. We also assumed that all tests results are negative because the health offices do not cancel the quarantine if at least one person is tested positive. However, this could change in future due to increasing vaccination numbers and one or two positive tested persons could be acceptable. In this case, a more precise estimation of tested specificity and, consequently, the false positive tested rate is needed to have a realistic model.

## 5. Conclusions

In this work, we introduced a statistical model for estimating the risk of an early group quarantine cancellation. Therefore, a day specific sensitivity and a specificity of 100% of the PCR-test were considered for the likelihood distribution. Moreover, the Beta-binomial distribution was chosen as prior for the transmission of the COVID-19, and a method for determining its parameters for specific situations was proposed. The whole model can be specified for any group event in any country. In this work, it was used for obtaining a prior distribution for school classes based on data obtained from literature. Based on the obtained prior distribution, we analyzed the usage of our statistical model for a decision support for quarantine cancellation and tested it on a real-world dataset of a German school class. Offered as a web application or integrated in a software solution, our statistical model could be used by employees of German health departments and could facilitate their daily work. A prototypical version of the web application (in German) is available upon request to the corresponding author.

In addition, we illustrated a decision rule based on the arbitrary threshold of 90% for the posterior probability that no transmission occurred. However, it is not clear, if this is the best rule for making a decision. The threshold could be also set higher, e.g., $p_{0}>0.95$, to reduce the remaining risk. Future work should address the choice of the threshold in dependence of factors, such as available test capacities. Moreover, we will implement other circumstances of the contact event (location, interactions, etc.) in the model and will apply it for other specific scenarios. In a next step, the benefit of using our tool in routine cases has to be evaluated. For this purpose, a broad evaluation study with German health departments is planned.

## Figures and Tables

**Table 1:** Contact categories defined by the RKI.

Category	Infection Risk
1.	high, close contact to infectious person
2.	low, sparse contact to infectious person
3.	medical staff with adequate protection

**Table 2:** A dataset of a school class from the health department in Berlin-Reinickendorf, which contains the last contact date, the PCR test date, and the PCR results.

Case Number	Date of Last Contact	PCR Test Date	PCR Test Result
1	10 August 2020	—	—
2	10 August 2020	—	—
3	10 August 2020	—	—
4	10 August 2020	18 August 2020	negative
5	10 August 2020	19 August 2020	negative
6	10 August 2020	19 August 2020	negative
7	10 August 2020	19 August 2020	negative
8	10 August 2020	20 August 2020	negative
9	10 August 2020	19 August 2020	negative
10	10 August 2020	19 August 2020	negative
11	10 August 2020	19 August 2020	negative
12	10 August 2020	19 August 2020	negative
13	10 August 2020	19 August 2020	negative
14	10 August 2020	19 August 2020	negative
15	10 August 2020	19 August 2020	negative
16	16 August 2020	19 August 2020	negative
17	18 August 2020	24 August 2020	negative

## Data Availability

The data that support the findings of this study are available from the corresponding author, upon reasonable request.
